# Incidence of Perfluoroalkyl Substances in Commercial Eggs and Their Impact on Consumer’s Safety

**DOI:** 10.3390/foods12203846

**Published:** 2023-10-20

**Authors:** Maria Nobile, Francesco Arioli, Dalia Curci, Claudia Ancillotti, Giulia Scanavini, Luca Maria Chiesa, Sara Panseri

**Affiliations:** 1 Department of Veterinary Medicine and Animal Science, University of Milan, Via dell’Università 6, 26900 Lodi, Italy; maria.nobile1@unimi.it (M.N.); francesco.arioli@unimi.it (F.A.); luca.chiesa@unimi.it (L.M.C.); sara.panseri@unimi.it (S.P.); 2 Biochemie Lab S.r.l., Via Limite, 27/G, 50013 Campi Bisenzio, Italy; c.ancillotti@biochemielab.it (C.A.); g.scanavini@biochemielab.it (G.S.)

**Keywords:** food safety, eggs, PFASs, HPLC-HRMS, risk characterisation, consumer’s safety, perfluoroalkyl substances

## Abstract

Eggs play an important role in a balanced diet; however, the European Food Safety Authority (EFSA) recognizes eggs as a major source of poly and per-fluoroalkyl substances (PFASs). In this study, the presence of PFASs was analysed in eggs produced by hens from Northern Italian regions, a PFASs-contaminated area. Sixty-five samples were analysed by high-performance liquid chromatography coupled with high-resolution mass spectrometry. The greatest presence of PFASs was found in eggs from Veneto and Emilia Romagna, and the most detected PFASs were perfluorobutanoic acid (PFBA) and perfluorooctanesulfonic acid (PFOS) (mean concentrations 0.30 ± 0.15 and 0.05 ± 0.00 ng g^−1^). Considering the most recent updates for the sum of the main four PFASs, the highest concentration found in the analysed samples was 0.05 ng g^−1^, well below the maximum limit set by the European Union. The PFAS intake evaluation confirmed that egg consumption does not represent a risk for Italian consumers.

## 1. Introduction

As a consequence of environmental pollution caused by industrial development and new agricultural practices, an increasing number of food-safety problems are leading governments to improve their efforts to better ensure food safety and consumer health [1]. Several toxicant residues can be detected in foods and along the food chain attempt human health [2]. Food may be contaminated at various stages of its production due to agricultural or farm practices, during the packaging process, or transport and storage [1]. 

The most sought-after categories of emerging contaminants in food are poly- and per-fluoroalkyl substances (PFASs), currently of extreme concern due to the recent update of regulations [3]. PFASs are a large group of organic compounds characterized by the presence of C-F bonds, well-known as very strong bonds, which provide them important chemical properties and environmental stability [4].

Since the 1950s, due to their properties, PFASs have been produced and used in several industrial applications to obtain fat- and water-resistant products [5,6,7]. 

PFASs are compounds capable of accumulating in animal and human blood and tissues, where they exert their toxicity as endocrine disruptors [8]. Particularly, PFASs affect the reproductive system, thyroid gland function, bone metabolism, and cause crucial health consequences on the immune and nervous systems [8]. 

The European Food Safety Authority (EFSA) scientific reports indicate that human exposure to PFASs may result from the consumption of contaminated food, beverages, and inhalation [9,10]. In 2020, EFSA established a tolerable weekly intake (TWI) of 4.4 ng kg^−1^ bw per week as the sum of the following four major PFASs: perfluorooctanoic acid (PFOA), perfluorooctanesulfonic acid (PFOS), perfluorononanoic acid (PFNA), and perfluorohexane sulphonate (PFHxS) based on the observation that these four PFASs most contribute to the levels observed in human serum [11]. The EFSA scientific evaluation of the risks to human health related to the presence of PFASs in food exhibited that eggs and egg products, along with fish meat and fruit and fruit products, contributed the most to the exposure [11]. Thus, the European Union (EU), to announce and protect food safety, has adopted Regulation 2022/2388, which has taken reaction from 1 January 2023. Regulation 2022/2388 provides the setting of maximum levels for four PFASs individually (PFOA, PFOS, PFNA, and PFHxS) and their total sum in fish muscle, meat, and eggs [3]. 

Recent information on Italian egg consumption is provided by the “Istituto di Servizi per il Mercato Agricolo Alimentare” (Ismea, Agricultural Food Market Services Institute) report (2021) related to 2020 [12]. The annual consumption of Italian eggs appears to be equal to 13.8 kg per capita, considering eggs and egg products, 60% of which is related to the consumption of eggs, which means that each person consumed 8.28 kg of eggs in 2020. As previously discussed, eggs could be a possible source of contaminants through several pathways, and in the scientific literature, some studies have been carried out to detect contaminants that may be involved in the egg production process. The scientific literature toward PFASs in eggs and derived products are summarized and presented in Table 1.

The present study aimed to investigate the presence of PFASs in eggs from hens raised in Northern Italy, an area known to be PFASs contaminated [13]. Moreover, for consumer protection, it was assessed whether the PFAS concentrations found in the eggs complied with the recent limits defined by the EU. The study involved eggs produced by different breeding systems, such as caged and free-range hens, to analyse whether different conditions and breeding behaviour could affect the potential contamination of chicken eggs differently [14,15], linking this information with the geographical origin of the eggs. 

Finally, the PFAS evaluation intake was carried out to compare the results obtained with the TWI suggested by EFSA.

**Table 1:** State of the art in the detection of PFAS in foods of animal and plant origin.

Reference	Analytes	Matrix	Extraction Technique	Instrumental Analysis	Limits of the Method (ng g^−1^)	Application Range Conc. (ng g^−1^)
[16]	PFOS, PFHxS, PFBS, PFOSA, PFNA, PFOA, PFHxA, PFHpA, PFUnDA, PFDA, PFDoDA	Pooled and yolk chicken egg form local markets	Homogenization and extraction with TBA, sodium carbonate buffer (pH 10) and MTBE. Centrifugation, pour 4 mL of the extract in a tube and perform the extraction again. Purification with SPE Oasis WAX cartridge. Evaporation and injection.	LC-MS/MS ^1^	LOQ ^2^ = 0.01 − 0.08	<LOQ − 87.6
[17]	PFOA and PFOS	Human milk, fish, meat, milk dairy products, cereal-based food, eggs, vegetables, honey and beverages	Homogenization, extraction with 1 mL of 0.5 M of TBA, 2 mL of sodium carbonate buffer (0.25 M, pH 10) and 5 mL of MTBE, mix and centrifugation. Do the step twice. Evaporation, resuspension, filtration and injection	LC-MS/MS	LOD ^3^ = 0.50	<LOD
[18]	FOSA, PFBA, PFPeA, PFHxA, PFHpA, PFOA, PFNA, PFDA, PFUdA, PFDoA, PFTrDA, PFTeDA, PFHxDA, PFODA, PFBS, PFHxS, PFOS, Br-PFOS, PFDS, PFHxPA, PFOPA, PFDPA	Meat, seafood, fish, milk, dairy products, and hen eggs	Homogenization, extraction with water, acetonitrile, and formic acid. QuEChERS extraction procedure was performed. Evaporation, resuspension, filtration, and injection	LC-MS/MS	MQL ^4^ = 0.001 − 0.01	<MQL − 1.96
[15]	PFHxA, PFHpA, PFOA, PFNA, PFDA, PFUnA, PFDoA, PFBuS, PFHxS, PFHpS, PFOS	Home produced and commercially produced eggs (organic, battery and free-range eggs)	Homogenization, fortification, and extraction with 10 mL of MeOH. Acidification and centrifugation. Purification with SPE Oasis WAX cartridge. Evaporation, resuspension, and injection.	LC-MS/MS	LOD = 0.15 LOQ = 0.5	<LOQ − 31.2
[19]	(PFOS, PFOA, PFNA, PFHxS	EBC, eggs from backyard chickens	Digestion with a sodium hydroxide solution, homogenization, addition of methanol and HCl (37%), centrifugation, addition of ultrapure water, purification by SPE Oasis WAX cartridges, resuspension	UPLC–MS/MS ^5^	LOD = 0.10 LOQ = 0.25	<LOQ − 3.5
[20]	PFSAs, PFBS, PFHxS, PFOS, PFDS, PFCAs, PFBA, PFPeA, PFHxA, PFHpA, PFOA, PFNA, PFDA, PFUnDA, PFDoDA, PFTrDA and PFTeDA, NaDONA, GenX	Home—produced eggs	Homogenization, add 10 mL of acetonitrile, shake. Centrifugation and not complete evaporation of the supernatant. Purification with graphitized carbon powder (Supelclean ENVI-Carb). Adding acetonitrile. Evaporation, resuspension, and injection.	UPLC-MS/MS	LOQ = 0.08 − 2.5	<LOQ − 241
[21]	PFBA, PFPeA, PFHxA, PFHpA, PFOA, PFNA, PFDA, PFUnDA, PFDoDA, PFBS, PFPeS, PFHxS, PFHpS, PFOS	Eggs from cage, ecological and free-range hens	Lyophilization, extraction with 10 mL of 0.01 M methanol/potassium hydroxide. Purification with Oasis WAX (150 mg, 6 mL) SPE cartridge and ENVI Carb SPE (500 mg, 6 mL). Evaporation, resuspension, and injection.	LC-MS/MS	LOQ = 0.005 − 0.163	<LOQ − 0.74
[22]	PFPeA, PFHxA, PFHpA, PFOA, PFNA, PFDA, PFUnDA, PFDoDA, PFTrDA, PFTeDA, PFBS, PFPeS, PFHxS, PFHpS, PFOS, PFNS, PFDS, PFDoDS	Commercial eggs (barn, organic and caged hen eggs)	Homogenization, extraction with 10 mL water and 10 mL acetonitrile. QuEChERS extraction (EN Method), mix and centrifugation. After drying 5 mL of the supernatant and adding 0.25 mL of 1% acetic acid in methanol, solid phase extraction (SPE) was carried out	UHPLC-HRMS ^6^	LOD = 0.0050 − 0.036 LOQ = 0.050	<LOQ − 0.042

^1^ LC-MS/MS = liquid chromatography—tandem mass spectrometry; ^2^ LOQ = limit of quantification; ^3^ LOD = limit of detection; ^4^ MQL = method quantification limit; ^5^ UPLC–MS/MS = ultra pressure liquid chromatography—tandem mass spectrometry; ^6^ UHPLC-HRMS = ultra high-pressure liquid chromatography—tandem high-resolution mass spectrometry.

## 2. Materials and Methods

### 2.1. Chemical and Reagents

The following perfluorinated carboxylates and sulphonates compounds were purchased from Chemical Research 2000 Srl (Rome, Italy): perfluorobutanoic acid (PFBA), perfluoropentanoic acid (PFPeA), perfluorohexanoic acid (PFHxA), perfluorobutane sulphonic acid (PFBS), perfluoroheptanoic acid (PFHpA), PFOA, PFHxS, PFNA, perfluorodecanoic acid (PFDA), PFOS, perfluorododecanoic acid (PFDoA), perfluoroundecanoic acid (PFUnDA), perfluorotridecanoic acid (PFTrDA), perfluorotetradecanoic acid (PFTeDA), perfluorohexadecanoic acid (PFHxDA), perfluorooctadecanoic acid (PFODA) and the two 13Clabeled internal standards (ISs) perfluoro-[1,2,3,4,5-13C5] nonanoic acid (MPFNA) and perfluoro-[1,2,3,4-13C4] octanesulfonic acid (MPFOS) were purchased from purchased from Chemical Research 2000 Srl (Rome, Italy). Analytical liquid chromatography-mass spectrometry (LC-MS) solvents and reagents were obtained from Merck (Darmstadt, Germany). Strata PFAS (WAX/GCB), 200 mg/50 mg/6 mL (purification cartridges) were provided by Phenomenex (Torrance, CA, USA).

### 2.2. Sample Collection

The 65 commercial eggs were analysed from Northern Italian markets. The eggs were from hens raised in farms located in Lombardy, Veneto, Emilia Romagna, Piedmont, and Friuli Venezia Giulia. In addition to differences related to provenance, the eggs under study also differed according to the type of farming. The eggs studied were from cage farms (6 eggs) and the following 3 different types of free-range systems: organic (21 eggs), indoor free-range (29 eggs), and outdoor free-range (8 eggs); meanwhile, for 1 egg was not possible to know the breeding system. The previous information is summarized in Table S1 and provided in the Supplementary Materials. 

As reported by Naing et al., 2006 [23], the following formula was used to verify whether the sample size was satisfactory:

N = Z^2^ × [P × (1 − P)]/D^2^, (1)

where Z has a value of 1.96 for a confidence limit of 95%, P is the expected prevalence and D is the precision of the estimate.

### 2.3. Sample Size

The 65 egg samples were provided, and to verify the suitability of the sample size, we used the formula reported in Section 2.2. The confidence limit was set at 95%, and the prevalence was set at 0.5 (50%), which allowed obtaining the highest size value for a given precision. This conservatory approach permitted to consider satisfactory a precision value of 12%. When there is a high value of the population, this formula makes it possible to calculate the sample size regardless of the population size. Considering an industrial egg production of 12.6 billion in Italy (2022), the obtained value can be considered satisfactory. 

### 2.4. Standard Solutions

Perfluoroalkyl substance stock solutions (1 mg mL^−1^) and working solutions (10 and 100 ng mL^−1^) were prepared in methanol and kept at −20 °C. 

### 2.5. PFAS Extraction Protocol

The extraction protocol was carried out following the procedure of our previous work [24]. Briefly, 5 g of homogenized sample were spiked with internal standards at 5 ng g^−1^, extracted with 10 mL of acetonitrile, vortexed, sonicated, and centrifuged (2500× *g*, 4 °C, 10 min). The dried supernatant was resuspended in 5 mL of water and purificated by STRATA PFAS cartridges. The final extract was resuspended in 200 µL 20 mM MeOH: ammonium formate (20:80 *v*/*v*), and eventually centrifuged in an Eppendorf tube if turbid. 

### 2.6. LC-HRMS Analysis

The analysis was carried out by a Vanquish (Thermo Fisher Scientific, Waltham, MA, USA) coupled to a Thermo Orbitrap™ Exploris 120 (Thermo Fisher Scientific, Waltham, MA, USA), using a HESI (heated electrospray ionization) source in negative ionization mode. For separation, a Raptor ARC-18 5 µm, 120 × 2.1 mm column (Restek, Bellefonte, PA, USA) was used. Furthermore, in order to retard PFASs already present in the system, a small Megabond WR C18 column (5 cm, 4.6 mm, i.d. 10 mm) was introduced before the injector. The chromatographic conditions and HRMS parameters are reported in our previous work [24]. The software used was Xcalibur ^TM^ 4.5 (Thermo Fisher Scientific, Waltham, MA, USA).

### 2.7. Parameter Validation Method

Validation was carried out according to SANTE/11312/2021 [25] and was clearly described by Nobile et al., 2023 [26]. The validation parameters of the 17 investigated PFASs are reported in Table 2. Briefly, the selectivity of the method was evaluated by injecting extracted blank egg samples, and the lack of signal near the expected PFAS retention time with a signal-to-noise ratio (S/N) < 3 indicated the absence of interference. 

The lowest spiking level (5 repetitions) with an S/N of at least 10, recovery between 70% and 120%, and relative standard deviation (RSD) of 20% was chosen as the limit of quantification (LOQ) (European Commission, 2021). The LOQs were in the range of 0.05–0.1 ng g^−1^ demonstrating high method sensitivity. The linearity was evaluated by 5-point calibration curves in solvent in the analytical range from LOQ to 10 ng g^−1^ for all analytes. Matrix-matched calibration curves created for 5 calibration points in duplicate were in the range from LOQ to 10 ng g^−1^. Both for the instrumental linearity and matrix calibration curves were obtained correlation coefficients higher than 0.99 for all compounds, indicating a satisfactory fit according to European validation criteria as well. The intra-day and inter-day precision, expressed a CV% (coefficient of variation), were assessed by 6 replicated on the same day and by 6 replicates in 3 distinct days, respectively, and were lower than 20% in agreement with the indicated tolerances for the validation criteria. The recovery, evaluated by comparing the concentrations of PFASs spiked before with those at the end of the extraction procedure, ranged from 70 to 116%, revealing good efficiency of the analytical protocol. The peak areas of the standards in a neat solution mix and the peak areas of PFASs injected following the extraction of a blank sample were compared to calculate the matrix effect, which exhibited a lower influence (<20%), with a percentage variation from 90% to 104%. 

### 2.8. Dietary Intake Estimation

The estimated daily intake (EDI) of PFASs was calculated as follows:

EDI = C × DC/BW,(2)

where C is related to the maximum sum of the four main PFASs found in the analysed eggs and DC is the daily egg consumption per capita in Italy divided by the consumer body weight (considering an average weight of 70 kg).

## 3. Results and Discussion

### 3.1. Occurrence of PFASs in Eggs

**Figure 1:**
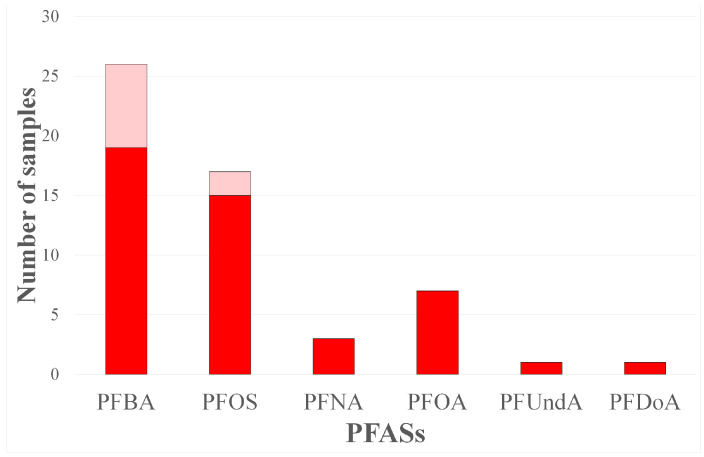
Number of samples in which PFASs have been detected. The full red part of the histograms indicates the number of samples in which PFASs have been detected with a value lower than the LOQ on the total of each group. Meanwhile, the light red indicates the ones with a value higher than the LOQ.

Only the following six PFASs were detected in the samples: PFBA, PFOS, PFNA, PFOA, PFUndA, and PFDoA. Contaminant concentrations were often below the quantification limits (Figure 1). Particularly, the mean values (considering only the samples where PFASs have been detected and quantified) were 0.30 ± 0.15 ng g^−1^ (19 samples), and 0.05 ± 0.00 ng g^−1^ (2 samples) for PFBA and PFOS, respectively. Meanwhile, in 3, 7, 1, and 1 samples PFNA, PFOA, PFUndA, and PFDoA were detected under the LOQ, respectively. The European Regulation 2022/2388 set the maximum limits (ML) in eggs equal to 1.0, 0.30, and 0.70 ng g^−1^ for PFOS, PFOA, and PFNA, respectively. The values detected in the samples analysed in the present study were well below the ML value set from the UE. In addition, as suggested by the European Regulation 2022/2388, considering the sum of the concentrations of the three detected PFASs in two egg samples the total concentrations were 0.05 ng g^−1^, “the lower bound concentrations are calculated on the assumption that all values below the quantification limit are zero” [3]. 

In the present study, to evaluate PFAS dietary intake, we considered the non-quantifiable values below the LOQ as half of the LOQ. In this scenario, the sum of the three PFASs detected in the two samples was 0.1 ng g^−1^.

**Figure 2:**
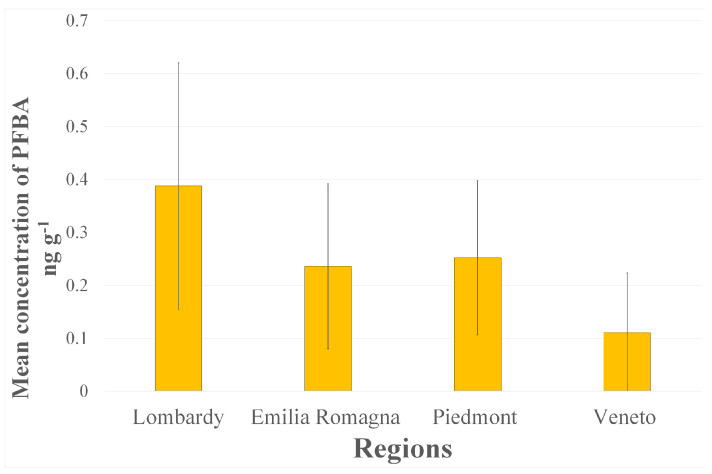
Mean concentration of PFBA (ng g^−1^) in eggs samples analysed in this study.

Notably, among the analytes found, there are both short- and medium-chain carboxylic compounds (PFBA and PFOA) and long-chain perfluorocarboxylic acids (including PFNA, PFUndA, and PFDoA), as well as perfluorosulfonic compounds (PFBS and PFOS). In the present study, the most detected PFAS were PFBA (40%) (Figure 2) and PFOS (26%). There was a higher incidence of long-chain PFAS, which is in according with the scientific literature. Some studies have reported that it is easier for long-chain analytes to bioaccumulate in eggs [27,28].

#### 3.1.1. PFAS Detection and Samples Provenience

**Figure 3:**
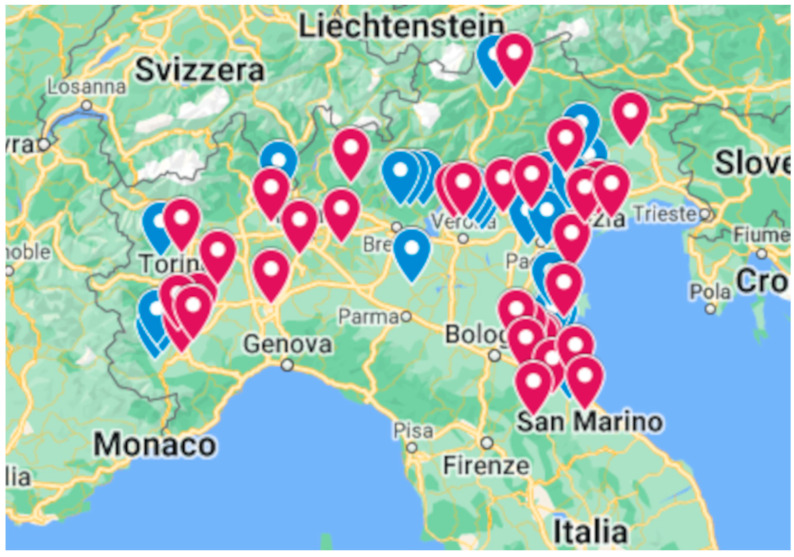
Map of the sites in which samples have been collected. They are reported as a red pointer where PFASs have been detected and with a blue one for the opposite ones.

**Figure 4:**
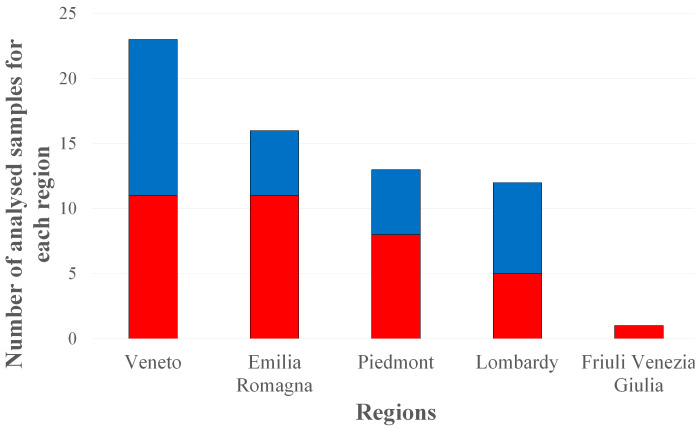
Number of samples collected in each region. In red the samples in which PFASs have been detected on the total of each group, instead in blue those in which PFASs were not present.

Considering the samples related to their origin, as shown in Figure 3 and Figure 4, it can be noted that the greatest presence of PFASs was found in eggs from farms in Veneto and Emilia Romagna, but the widest variety of PFASs was found in samples from Lombardy. 

The World Health Organization (WHO) studied the Veneto region, particularly regarding the industrial activities in the area that had polluted the water resources, both surface waters and groundwater, of the region. Moreover, as reported by the WHO, studies conducted by the Italian Ministry of Environment, Land, and Sea reported that of all the European rivers studied, the Po River had the highest concentrations of PFOA [13]. The Po River crosses the Piedmont, Lombardy, Veneto, and Emilia Romagna regions, which show the greatest evidence of PFASs in the samples.

PFASs were found in 11 out of 23 eggs from Veneto; the analytes found were PFBA and PFOS. A report of the Veneto Region on the monitoring of contamination in water for human consumption reported the province of Verona as the area with the greatest impact of pollution by perfluoroalkyl substances, relegating the province of Venice to a green zone and, therefore, relatively safe [29]. In the present study, PFASs were found in eggs from farms located in the entire Veneto area, not only in the Verona area but also in the Venice surroundings. This underlines the need for improved control. Furthermore, the last November 2022 survey of the population in the Veneto region showed a serum concentration of 2.8 ng mL^−1^ for PFOS [30].

PFASs were found in 11 out of 16 eggs from Emilia Romagna. The analytes detected in these eggs were PFOS, PFOA, PFBA, and PFNA. Emilia Romagna is an Italian region crossed by the Po River that is polluted by PFASs [13], but this region is also located near the Adriatic Sea, which is highly polluted by PFASs, especially PFOA, PFOS, and PFHxS [31]. 

For Piedmont, PFASs were found in 8 out of 13 eggs. In particular, PFOS, PFOA, and PFBA were detected. In Lombardy, PFASs were found in five out of twelve eggs analysed. Specifically, the PFASs detected were PFBA, PFOS, PFOA, PFNA, PFUndA, and PFDoA. Only one egg from Friuli Venezia Giulia was analysed, and PFBA was found to be lower than the LOQ.

#### 3.1.2. Comparison between PFAS Levels in Eggs from Different Countries

The scientific literature shows only two studies related to the analysis of PFASs in commercial eggs carried out in Italy. Guerranti et al., 2013 [17] analysed PFOS and PFOA levels of commercial eggs in four pooled samples. More recently, Chiumiento et al., 2023 [22] analysed PFASs in eggs from the Italian market and showed the presence of only long-chain PFASs (PFHpA, PFNA, PFDA, PFDoDA, PFHxS, PFOS, and perfluorododecane sulfonic acid (PFDoDS)), in which, only seven analytes were quantified above the LOD. Regarding them, PFHpA, PFNA, PFDA, PFOS, and PFDoDS were found in organic eggs; PFNA, PFDA, PFDoDA, and PFHxS were found in caged eggs; PFHpA, PFNA, PFFDA, PFHxS, and PFOS were found in barn eggs. The study did not find differences in PFAS quantification based on the farm type. The eggs from hens raised through different types of farming were analysed in the present study, but no trends could be shown based on this factor. In fact, for each type of rearing in at least half of the samples, PFASs were detected. This suggests that the contamination is not attributable to the type of rearing, but rather to the environment in which the hens are raised or the feed they are fed. 

In the study of Mikolajczyk et al., 2022 [21] on 45 egg samples from organic (n = 15), free-range (n = 15), and battery cage (n = 15) hens collected in Poland, the 4 main PFASs monitored, PFOS, PFOA, PFNA, and PFHxS, were quantifiable only in organic and free-range eggs, with the highest concentrations in organic eggs, although the differences between these two groups were not statistically significant. EFSA reports that the European average concentration of PFOS is 0.27 μg kg^−1^ w. w. [11], Mikolajczyk et al., 2022 [21] found 0.13 μg kg^−1^ w. w. as the highest PFOS concentration, which is in line with what was reported for Norway by Hlouskova et al., 2013 [18] 0.12 μg kg^−1^ w. w., i.e., lower than the European average. In contrast, differences are reported for Italian backyard hens (0.49–0.79 μg kg^−1^ w. w. [19]), for China (34.7–107 μg kg^−1^ w. w.; [16]), and from data from the Netherlands (median μg kg^−1^ w. w., on egg yolk) and Greece (median of 3.5 and 1.1 μg kg^−1^ w. w., on egg yolk, respectively; [15])). In the present study, the average PFOS concentration detected was 0.05 ± 0.00 ng g^−1^, which is lower than that reported by Gazzotti et al., 2021 [19]; however, this study was conducted on backyard hens, which are more prone to possible PFAS contamination. 

The European average concentration for PFOA reported by EFSA Is 0.11 μg kg^−1^ w. w. [11], in the study by Mikolajczyk et al., 2022 [21] it is instead 0.046 μg kg^−1^ w. w. The value found by Mikolajczyk et al., 2022 [21] is five times higher than the value reported by [19] for Italy (0.01 μg kg^−1^ w. w.). Data from the present study and Mikolajczyk et al., 2022 [21] agree with the concerns regarding PFHxs and PFBA. In fact, PFHxS was also not found in any samples in the study by Mikolajczyk et al., 2022 [21], and this agrees with the European data reported by EFSA, i.e., 0.000–0.06 μg kg^−1^ w. w. 

About the determination of PFBA, in Mikolajczyk et al., 2022 [21] it was found in all types of eggs, with a mean concentration for all production types of 0.24, 0.25, and 0.27 μg kg^−1^ w. w., which, in turn, agrees with data from Belgium (0.099 μg kg^−1^ w. w.) and Czech (0.099 μg kg^−1^ w. w.). Table 3 reports the PFASs detected in samples from all the investigated regions.

#### 3.1.3. Further Considerations

Eight eggs analysed in this study were from chickens fed with an all-vegetable diet; in particular, the feeding was free of animal fats and meals and enriched with flaxseed, cereals, and legumes. PFASs were detected in two out of these eight eggs. The detected PFASs were PFBA and PFOS, with values of 0.39 ng g^−1^ and below the LOQ, respectively. Information regarding hen feeding used in this study made it possible to speculate on the topic. Fish meal is a powdery substance prepared from fish and fish trimmings, generally identified as the main feed in hen farming systems [32], and it is also known to be an important source of PFASs [33]. Therefore, it is possible to assume that some of the analytes found in eggs are related to hen feeding; however, PFASs have also been found in the eggs of hens receiving an all-vegetable diet. This may support the idea that PFAS contamination originates from the environment, especially in animal drinking water [34]. 

### 3.2. Human Intake of PFASs through Diet

Information on Italian egg consumption provided by an Ismea report (2021) led to a clear consumption of 159 g and 23 g of eggs consumed weekly and daily, respectively [12]. As suggested by EFSA, the intake evaluation was conducted considering the cumulative sum of the four main PFASs (PFOA, PFOS, PFNA, and PFHxS). In particular, the intake was calculated, considering the eggs with the higher amount of the suggested PFASs, which showed a sum of 0.1 ng g^−1^. Particularly, the considered eggs showed concentrations of 0.05, 0.025 and 0.025 ng g^−1^ PFOS, PFOA and PFNA, respectively, whereas PFHxS was not detected.

It is mandatory to underline that the last European Regulation reporting maximum limits for PFASs in several matrices suggests considering equal to 0 a detected value lower than the LOQ. To have a more conservative approach, we considered half of the LOQ a value lower than the LOQ. 

EFSA established a TWI of 4.4 ng kg^−1^ bw per week; PFAS daily intake (EDI) calculated is 0.033 ng kg^−1^, which means that the weekly intake found (0.23 ng kg^−1^) through the analysed samples represents only 5.23% of the tolerable intake.

This evaluation allows us to compare the TWI only with the PFASs that resulted from egg consumption. But, it is well known that our diet is constituted of different foods and beverages that can be a source of PFASs. Concerns regarding PFAS monitoring in drinking water are validated by the knowledge that drinking water pollution most influences PFAS serum levels [35]. According to Regulations, in January 2014, after the discovery of significant surface, subterranean, and drinking water contamination in the Veneto Region (Trissino, Vicenza province), the Italian National Health Institute (ISS) set the maximum levels for PFOS (≤30 ng L^−1^), PFOA (≤500 ng L^−1^), and other PFAS (≤500 ng L^−1^) [36]. Nowadays, for drinking water, the most recent Italian Legislative Decree n.18/2023, which has taken reaction from March 2023, established maximum limits of 0.5 and 0.1 µg L^−1^ for the “Total PFASs” (obtained considering the total sum of the detected PFASs) and “PFAS Sum” (obtained only considering the PFASs of greatest concern), respectively. Meanwhile, for other matrices of different food chains, Chiesa et al., 2022 [24] investigated the presence of PFASs in several fish species from the Mediterranean Sea and North Italian Lake, detecting the presence of PFBA, PFBS, and PFOS in the ranges of <LOQ − 26.48, <LOQ − 8.27, and <LOQ − 24.41 ng g^−1^, for lake fish respectively, and <LOQ − 17.99, <LOQ − 0.67 ng g^−1^ for PFBA and PFOS in sea fish, respectively. Furthermore, Barbarossa et al., 2014 [37] investigated the presence of PFASs in Italian cow milk, detecting PFOS and PFOA with maximum concentrations of 0.97 and 0.32 ng L^−1^, respectively. Herzke et al., 2013 [38] investigated the presence of PFASs in several vegetables coming from European Countries, including Italy, detecting PFHxA, PFHpA, PFOA, PFNA, PFDcA, PFUnA, PFDoA, PFOS in the concentration range from <MQL (method quantification limit) to 121 ng kg^−1^. Arioli et al., 2019 [39] investigated the presence of PFASs in the meat of game animal species from an Italian subalpine area, detecting PFOS in wild boars (1.44 ± 0.86 ng g^−1^). Their ubiquitary presence underlines the need to consider not only the single investigated matrix but the totality of beverages and foods daily consumed.

## 4. Conclusions

In the eggs analysed in the present study, 6 out of 17 PFASs were found, including both short- and medium-chain carboxylic compounds (PFBA and PFOA) and long-chain perfluorocarboxylic acids.

Considering the data on the detection of PFASs in eggs from northern Italian markets, the most detected ones were PFBA and PFOS, with the highest presence found in eggs from Veneto and Emilia Romagna. Their mean concentrations were 0.30 ± 0.15 and 0.05 ± 0.00 ng g^−1^, respectively, and their detection may be linked to PFAS environmental contamination. Despite this consideration, PFAS levels found in the eggs were below the MLs set by the EU. Furthermore, results could suggest the assumption that part of PFASs found in the eggs may result from the hens feeding, and additional studies are required to learn more and deepen the topic. 

Finally, PFAS intake through eggs does not seem to represent a risk for Italian consumers, considering that this food comes from areas recognized for pollution by PFASs.

## Figures and Tables

**Table 2:** Validation parameters of the 17 investigated PFASs.

Compound	LOQ (ng g^−1^)	Recovery %	CV Intra-Day %	CV Inter-Day %	Matrix Effect %	Regression Equation
PFBA	0.05	116	14	17	104	y = 0.0861x + 0.0075
PFPeA	0.05	109	10	13	102	y = 0.1134x + 0.0008
PFBS	0.05	105	9	14	102	y = 0.8533x − 0.0056
PFHxA	0.05	93	6	12	99	y = 0.1484x − 0.0003
PFHpA	0.05	89	7	11	98	y = 0.2789x + 0.0155
PFHxS	0.05	90	9	11	101	y = 0.9212x + 0.049
PFOA	0.05	104	6	10	99	y = 0.4157x − 0.001
PFNA	0.05	92	10	16	99	y = 0.2643x − 0.0217
PFOS	0.05	95	8	14	102	y = 0.6592x − 0.1527
PFDA	0.05	79	12	16	98	y = 0.32x − 0.0059
PFUnDA	0.05	80	10	13	98	y = 0.3929x − 0.1277
PFDS	0.05	90	10	14	96	y = 0.5704x − 0.0364
PFDoA	0.05	72	11	16	92	y = 0.2471x − 0.1115
PFTrDA	0.1	71	12	15	93	y = 0.1734x − 0.0102
PFTeDA	0.1	70	15	18	93	y = 0.1579x − 0.0126
PFHxDA	0.1	71	17	18	91	y = 0.0968x − 0.0163
PFODA	0.1	70	17	20	90	y = 0.0226x + 0.0025
C6O4	0.5	72	15	18	92	y = 0.1330 + 0.0113

**Table 3:** Concentration (ng g^−1^) of the six detected PFASs in all the samples divided by their geographical origin.

	PFBA	PFOS	PFNA	PFOA	PFUndA	PFDoA
Sample	Concentration (ng g^−1^)					
	**VENETO REGION**					
1	n.d. ^1^	<LOQ	n.d.	n.d.	n.d.	n.d.
2	n.d.	<LOQ	n.d.	n.d.	n.d.	n.d.
3	0.30	n.d.	n.d.	n.d.	n.d.	n.d.
4	n.d.	n.d.	n.d.	n.d.	n.d.	n.d.
5	0.21	<LOQ	n.d.	n.d.	n.d.	n.d.
6	n.d.	<LOQ	n.d.	n.d.	n.d.	n.d.
7	0.17	<LOQ	n.d.	n.d.	n.d.	n.d.
8	n.d.	n.d.	n.d.	n.d.	n.d.	n.d.
9	n.d.	n.d.	n.d.	n.d.	n.d.	n.d.
10	n.d.	n.d.	n.d.	n.d.	n.d.	n.d.
11	<LOQ	<LOQ	n.d.	n.d.	n.d.	n.d.
12	n.d.	n.d.	n.d.	n.d.	n.d.	n.d.
13	n.d.	n.d.	n.d.	n.d.	n.d.	n.d.
14	n.d.	n.d.	n.d.	n.d.	n.d.	n.d.
15	n.d.	n.d.	n.d.	n.d.	n.d.	n.d.
16	n.d.	n.d.	n.d.	n.d.	n.d.	n.d.
17	n.d.	n.d.	n.d.	n.d.	n.d.	n.d.
18	<LOQ	n.d.	n.d.	n.d.	n.d.	n.d.
19	<LOQ	n.d.	n.d.	n.d.	n.d.	n.d.
20	<LOQ	n.d.	n.d.	n.d.	n.d.	n.d.
21	n.d.	<LOQ	n.d.	n.d.	n.d.	n.d.
22	n.d.	n.d.	n.d.	n.d.	n.d.	n.d.
23	n.d.	n.d.	n.d.	n.d.	n.d.	n.d.
	**EMILIA ROMAGNA REGION**					
24	0.21	<LOQ	n.d.	n.d.	n.d.	n.d.
25	n.d.	<LOQ	n.d.	n.d.	n.d.	n.d.
26	0.23	<LOQ	n.d.	n.d.	n.d.	n.d.
27	n.d.	n.d.	n.d.	n.d.	n.d.	n.d.
28	0.42	n.d.	n.d.	n.d.	n.d.	n.d.
29	n.d.	<LOQ	<LOQ	<LOQ	n.d.	n.d.
30	n.d.	n.d.	n.d.	<LOQ	n.d.	n.d.
31	n.d.	n.d.	n.d.	n.d.	n.d.	n.d.
32	0.41	n.d.	n.d.	n.d.	n.d.	n.d.
33	n.d.	n.d.	n.d.	n.d.	n.d.	n.d.
34	n.d.	n.d.	n.d.	n.d.	n.d.	n.d.
35	0.12	n.d.	n.d.	n.d.	n.d.	n.d.
36	n.d.	<LOQ	n.d.	n.d.	n.d.	n.d.
37	n.d.	n.d.	n.d.	n.d.	n.d.	n.d.
38	<LOQ	0.5	<LOQ	<LOQ	n.d.	n.d
39	n.d.	<LOQ	n.d.	<LOQ	n.d.	n.d.
	**LOMBARDY REGION**					
40	n.d.	n.d.	n.d.	n.d.	n.d.	n.d.
41	n.d.	n.d.	n.d.	n.d.	n.d.	n.d.
42	0.44	0.05	<LOQ	<LOQ	<LOQ	<LOQ
43	n.d.	n.d.	n.d.	n.d.	n.d.	n.d.
44	n.d.	n.d.	n.d.	n.d.	n.d.	n.d.
45	0.39	n.d.	n.d.	n.d.	n.d.	n.d.
46	<LOQ	n.d.	n.d.	n.d.	n.d.	n.d.
47	n.d.	n.d.	n.d.	n.d.	n.d.	n.d.
48	n.d.	n.d.	n.d.	n.d.	n.d.	n.d.
49	n.d.	n.d.	n.d.	n.d.	n.d.	n.d.
50	0.67	n.d.	n.d.	n.d.	n.d.	n.d.
51	0.41	<LOQ	n.d.	<LOQ	n.d.	n.d.
	**PIEDMONT REGION**					
52	0.43	n.d.	n.d.	n.d.	n.d.	n.d.
53	0.28	n.d.	n.d.	n.d.	n.d.	n.d.
54	n.d.	<LOQ	n.d.	<LOQ	n.d.	n.d.
55	0.06	n.d.	n.d.	n.d.	n.d.	n.d.
56	n.d.	n.d.	n.d.	n.d.	n.d.	n.d.
57	n.d.	n.d.	n.d.	n.d.	n.d.	n.d.
58	0.25	n.d.	n.d.	n.d.	n.d.	n.d.
59	n.d.	n.d.	n.d.	n.d.	n.d.	n.d.
60	n.d.	n.d.	n.d.	n.d.	n.d.	n.d.
61	0.07	n.d.	n.d.	n.d.	n.d.	n.d.
62	0.29	n.d.	n.d.	n.d.	n.d.	n.d.
63	0.39	n.d.	n.d.	n.d.	n.d.	n.d.
64	n.d.	n.d.	n.d.	n.d.	n.d.	n.d.
	**FRIULI VENEZIA GIULIA REGION**					
65	<LOQ	n.d.	n.d.	n.d.	n.d.	n.d.

^1^ n.d. = not detected.

## Data Availability

The data used to support the findings of this study can be made available by the corresponding author upon request.

## Supplementary Materials

The following supporting information can be downloaded at: https://www.mdpi.com/article/10.3390/foods12203846/s1, Table S1: Samples information related to the geographical origin of the eggs, included the coordinates of the farm, and the type of breeding.
