# Supplementary material for: Incidence of Perfluoroalkyl Substances in Commercial Eggs and Their Impact on Consumer’s Safety
Source: Foods. 2023 Oct 20;12(20):3846. doi: 10.3390/foods12203846 (PMC10605999; doi:10.3390/foods12203846)
Supplement: Supplementary file 1 [file foods-12-03846-s001.zip › foods-2665029-supplementary.pdf]

Geographical origin	Coordinates	Type of breeding
**Lombardy**	45.511020741125094, 10.454968841610937	Outdoor free-range
**Lombardy**	45.511020741125094, 10.454968841610937	Caged
**Veneto**	45.96519359209739, 12.145086570093564	Indoor free-range
**Veneto**	45.24913426315191, 12.065908215993177	Organic
**Veneto**	45.59467315275481, 10.835991553417454	Organic
**Lombardy**	45.5970054722651, 9.661677854528628	Indoor free-range
**Veneto**	45.37744620512782, 11.709918168592175	Indoor free-range
**Emilia Romagna**	44.44679663767255, 11.86549205767098	Indoor free-range
**Emilia Romagna**	44.44679663767255, 11.86549205767098	Organic
**Emilia Romagna**	44.83493003054947, 12.090421788354897	Indoor free-range
**Veneto**	45.640555319566865, 11.419746303696861	Indoor free-range
**Lombardy**	45.07148842573035, 10.36837069556596	Outdoor free-range
**Lombardy**	45.511020741125094, 10.454968841610937	Organic
**Emilia Romagna**	44.11327258085736, 12.198174510524163	Indoor free-range
**Emilia Romagna**	44.495790061379616, 11.881550430129412	Organic
**Lombardy**	45.48550847047831, 9.318373774861373	Organic
**Piedmont**	44.4980288902129, 7.710632511645097	Caged
**Piedmont**	44.72305992423337, 7.9077746079482365	Indoor free-range
**Lombardy**	45.511020741125094, 10.454968841610937	Organic
**Emilia Romagna**	44.495790061379616, 11.881550430129412	Caged
**Piedmont**	44.6834840720654, 7.618793112739635	Indoor free-range
**Emilia Romagna**	44.44679663767255, 11.86549205767098	Outdoor free-range
**Lombardy**	45.07148842573035, 10.36837069556596	Organic
**Piedmont**	45.06458940230416, 8.124761919316073	Outdoor free-range
**Piedmont**	44.45547953562942, 7.399892912729884	Indoor free-range

**Piedmont**	44.6834840720654, 7.618793112739635	Indoor free-range
**Lombardy**	45.07148842573035, 10.36837069556596	Indoor free-range
**Emilia Romagna**	45.358155141869084, 11.325788348002545	Indoor free-range
**Veneto**	45.67342307699246, 11.754352174861372	Indoor free-range
**Emilia Romagna**	44.16457683837938, 11.999690658964717	Indoor free-range
**Piedmont**	45.07419713135141, 7.660166817190393	Organic
**Piedmont**	45.215081069004775, 7.5227526974206205	Indoor free-range
**Piedmont**	44.680710137286766, 7.615404012739527	Organic
**Emilia Romagna**	44.50735180134222, 12.031418253212722	Caged
**Lombardy**	45.63887749699925, 8.876600668603588	Organic
**Lombardy**	45.63944011782717, 8.876761603696863	Indoor free-range
**Emilia Romagna**	44.49102974270023, 11.880917426224933	Indoor free-range
**Piedmont**	44.697162293233596, 7.605779732532351	Organic
**Veneto**	46.50517285107982, 11.335188119038822	Indoor free-range
**Veneto**	46.50475929436053, 11.334930624464203	Organic
**Veneto**	45.386893614996005, 11.212742026263589	Organic
**Veneto**	45.396087724575345, 11.178423170441425	Organic
**Veneto**	45.430223170970045, 11.074597655822549	Indoor free-range
**Veneto**	45.430147857478815, 11.074737128113878	Indoor free-range
**Emilia Romagna**	43.77518084993647, 12.082337186506473	Outdoor free-range
**Piedmont**	44.9089744309376, 8.747418813493528	Indoor free-range
**Lombardy**	45.32122023661332, 9.074732273012936	Caged
**Piedmont**	44.65352375110166, 7.647804373012941	Organic
**Veneto**	45.35175307495007, 11.332621226437174	Organic
**Emilia Romagna**	44.113365093868474, 12.198174528835489	Outdoor free-range
**Emilia Romagna**	44.88357871964651, 11.976842055077254	Indoor free-range
**Veneto**	45.70921803798129, 11.946055095593707	Indoor free-range

**Piedmont**	44.68063385132885, 7.615361097456335	Organic
**Veneto**	45.66241244543971, 12.382769826275531	Outdoor free-range
**Veneto**	46.091285384864825, 12.17065891280082	Organic
**Veneto**	Not provided	Indoor free-range
**Emilia Romagna**	Not provided	Not provided
**Veneto**	45.598329727393256, 12.240045601848433	Outdoor free-range
**Veneto**	45.598329727393256, 12.240045601848433	Indoor free-range
**Friuli Venezia Giulia**	46.07171584269519, 13.018222055822546	Organic
**Veneto**	45.598329727393256, 12.240045601848433	Indoor free-range
**Veneto**	45.592059277963216, 12.121750726272513	Organic
**Veneto**	45.35599994439076, 12.00821432440254	Caged
**Emilia Romagna**	44.113365093868474, 12.198174528835489	Indoor free-range
**Veneto**	45.592059277963216, 12.121750726272513	Indoor free-range
